# Long-term survival is possible using cytoreductive surgery plus HIPEC for sarcomatosis—Case report of 2 patients

**DOI:** 10.1016/j.ijscr.2019.09.009

**Published:** 2019-09-21

**Authors:** Paul H. Sugarbaker

**Affiliations:** Program in Peritoneal Surface Malignancies, MedStar Washington Hospital Center, 106 Irving St., NW, Suite 3900, Washington, DC, 20010, USA

**Keywords:** Peritoneal metastases, Intraperitoneal chemotherapy, Doxorubicin, Cisplatin, Ifosfamide, Hyperthermia, EIPL (extensive intraperitoneal lavage), PCI (peritoneal cancer index), Case report

## Abstract

**Background:**

Sarcomatosis results from the dissemination of cells from an abdominal or pelvic primary sarcoma to the peritoneal surfaces. In most patients this is a lethal condition.

**Methods:**

The clinical and radiologic features of two patients with sarcomatosis were reviewed. Cytoreductive surgery and hyperthermic intraperitoneal chemotherapy (HIPEC) were used for treatment. Current status of these two patients was available.

**Results:**

These two patients had a large extent of disease. In one, a myxoid sarcoma filled the pelvis. In another, all quadrants of the abdomen and pelvis were involved by peritoneal metastases from a uterine leiomyosarcoma. The grade of these two tumors was moderate and low. Surgical resection of all sarcomatosis was performed and this was followed by HIPEC. Clinical and radiologic follow-up at 37 and 58 months after complete cytoreduction show no evidence of disease.

**Conclusion:**

An effort to identify patients with sarcomatosis who are predicted to have a complete cytoreduction should occur when these patients are evaluated. Patients with a complete resection and a sarcoma-specific HIPEC may have prolonged disease-free survival. No other treatments for these patients have been described.

## Highlights

• Sarcomatosis is the progression of visible malignant nodules on peritoneal surfaces.
• Long-term disease-free survival is unusual if high grade sarcomatosis is treated by CRS and HIPEC.
• In two patients prolonged disease-free survival has been observed with complete resection of low grade disease plus HIPEC.
• The lack of deep tissue invasion with low grade sarcoma may account for the long-term success of CRS and HIPEC.

## 1 Introduction

Metastases from abdominal or pelvic sarcomas are most commonly by vascular routes to liver with visceral sarcomas or to the lungs with retroperitoneal sarcoma. Metastases to lymph nodes are very unusual and if it occurs suggests a poor prognosis [1,2]. A second pattern of metastatic disease is to the abdominal or pelvic surfaces as sarcomatosis. With primary disease sarcomatosis is unusual occurring in approximately 10% of patients. However, in surgery for treatment of recurrent disease, sarcomatosis is present in a majority of patients [3,4]. The etiology for the high incidence of peritoneal metastases has not been definitively established but a spillage of cancer cells at the time of primary sarcoma resection is likely to be a frequent cause. This occurrence of sarcomatosis must be considered a prominent part of the natural history of these sarcomas. An option for treatment of peritoneal metastases in the absence of systemic metastases is complete surgical resection of local disease at the resection site and on peritoneal surfaces [5,6]. Long-term survival after reoperative surgery is estimated at 30%. In an attempt to improve the results of reoperation in patients with sarcomatosis, hyperthermic intraperitoneal chemotherapy [HIPEC] has been added to complete resection perhaps with some improvement [4,[7], [8], [9], [10], [11], [12], [13]]. But the possible benefits of the perioperative chemotherapy have never been confirmed by a trial. A randomized controlled trial of cytoreductive surgery alone versus cytoreductive surgery plus hyperthermic intraperitoneal cisplatin showed no benefit for the HIPEC [14]. These single institution studies confirm that some patients with recurrence confined to the abdomen and pelvis do profit from reoperative surgery. Selection factors for success with reoperation have not been defined. The grade of the sarcomatosis was not analyzed for its impact on outcome in these study of prognostic indicators for treatment of sarcomatosis. This manuscript focuses on two patients who have profited long-term from reoperation plus HIPEC for recurrent sarcoma. The clinical features of these two patients were carefully studied to determine which patients should be considered for reoperation. I suggest that the clinical features associated with long-term survival in these two patients are important in order to make sure patients who may profit are treated.

## 2 Materials and methods

Data on these two patients was prospectively recorded and then retrospectively reviewed at an academic institution. This research work has been reported in line with the SCARE criteria [15]. This study was registered as a case report on the www.researchregistry.com website with UIN 4889.

A HIPEC treatment was used in both patients using chemotherapy agents known to be active with soft tissue sarcoma. The HIPEC treatment was for 90 min with the intraperitoneal chemotherapy maintained at 41.0–42.5 °C by a hyperthermia pump. Chemotherapy agents were placed in an aqueous solution of 1.5% dextrose peritoneal dialysis solution with a total volume of 1.5 L/m^2^. Cisplatin was administered at 50 mg/m^2^ and doxorubicin at 15 mg/m^2^ in the HIPEC solution. Ifosfamide was given at 1300 mg/m^2^ as continuous infusion intravenously over the 90 min of HIPEC. In order to avoid uroendothelial damage from the ifosfamide, sodium-2-mercaptoethanesulfonate (MESNA) at 260 mg/m^2^ was administered intravenously 15 min before initiation of ifosfamide infusion, 4 h after and 8 h after. A forced diuresis of 100 ml of urine every 15 min was maintained during the HIPEC and for 90 min after completion of HIPEC.

## 3 Patient presentation

### 3.1 Patient 1

In August 2005, a 28-year-old woman upon routine Pap smear had abnormal findings. An ultrasound led to the identification of a left-sided pelvic mass. A surgical procedure resected the mass which upon histopathologic study showed myxoid liposarcoma.

In July 2007, a recurrence of the left-sided pelvic mass was diagnosed. A reoperative procedure resected this mass and the left adnexa.

In October 2015, the mass recurred in the pelvis with an extension to the retroperitoneum. A third surgery was performed and reported as a radical resection with clear margins.

**Fig. 1:**
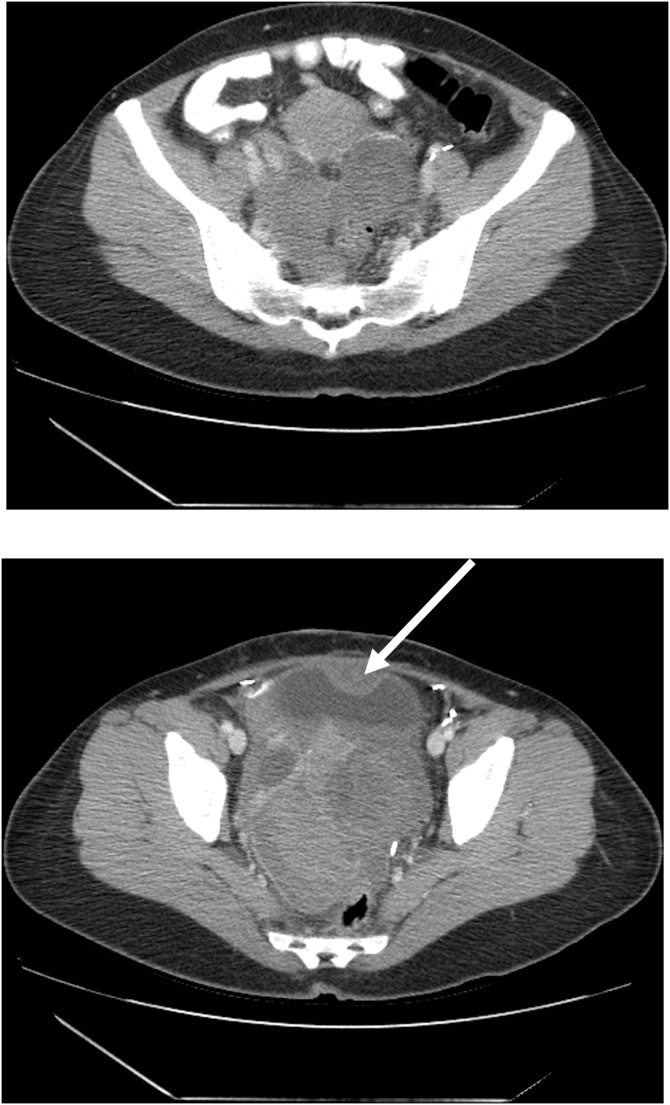
(top) CT shows a multilobulated mass greater on the left than the right. It is immediately adjacent to pelvic peritoneum on the right and covers the rectosigmoid junction on the left. It pushes the uterus superiorly. (bottom) CT shows a large mass that occupies the entire true pelvis. The rectum is markedly compressed. An arrow marks a separate sarcoma nodule at the dome of the bladder.

In March 2016, the patient returned with complaints of increasing pain in the left inner thigh. Bowel function continued but constipation was severe and increasing. CT was performed on February 20, 2016 (Fig. 1 top and bottom). A multilobular mass was present in the false pelvis, filled the true pelvis forcing the uterus and bladder superiorly, and markedly narrowed the rectum.

**Fig. 2:**
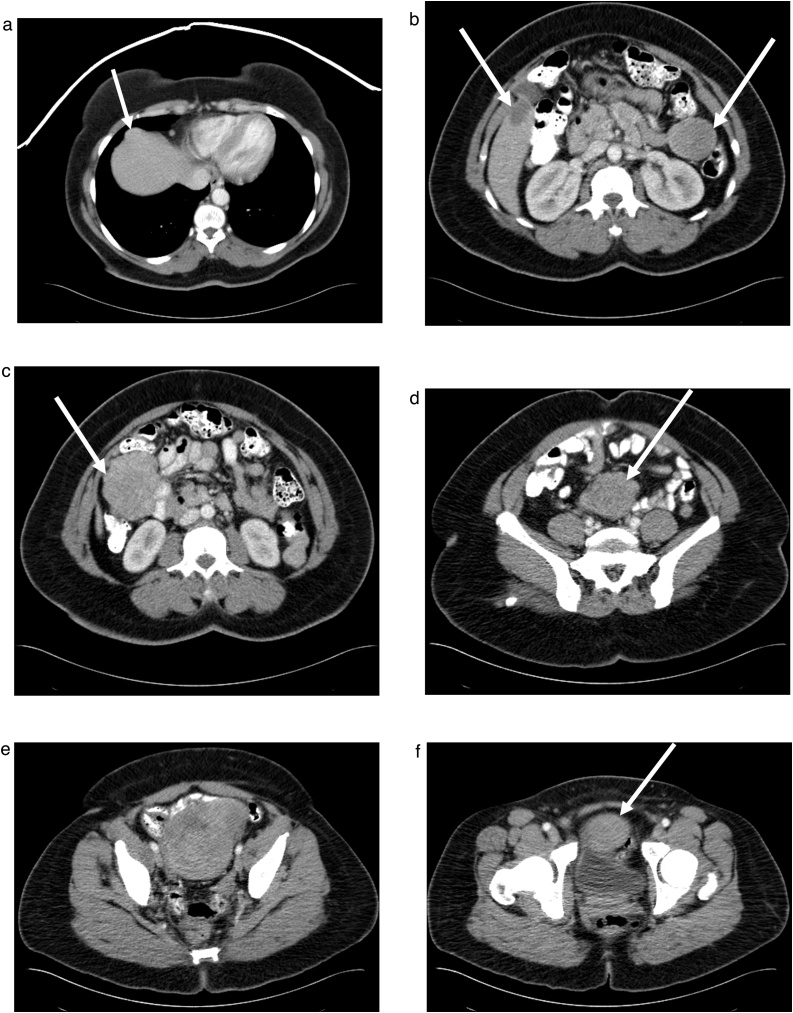
Specimen of pelvic peritoneum, uterus and right ovary, rectosigmoid colon and recurrent myxoid liposarcoma.

In March 22, 2016, the patient underwent a seven-hour cytoreductive surgery. Prior to opening the abdomen, bilateral ureteral stents were placed. Upon exploration, the multilobulated mass was attached to pelvic peritoneum, bladder, uterus and rectum. Peritoneal cancer index was estimated at 9 [16]. The en bloc resection began with a peritonectomy well beyond the mass itself. The visceral peritoneum from the bladder was resected using electroevaporative surgery because the sarcoma was pushing against this pelvic peritoneal surface [17]. An extraperitoneal hysterectomy was performed along with a rectosigmoid colon resection. The peritoneum above the cul de sac and pararectal fossae was removed intact with the specimen. Both ureters were stripped of adherent scar tissue especially on the left using electrosurgery but taking care not to damage the ureters with heat. The resected myxoid sarcoma, pelvic peritoneum, uterus with right ovary and tube and rectosigmoid colon were removed en bloc (Fig. 2). An isolated mass from the dome of the bladder was resected separately. The completeness of cytoreduction score was 0 [16]. Before performing the colorectal anastomosis and closing the abdominal incision, a warm chemotherapy wash of the entire abdomen and pelvis was performed using chemotherapy specially selected for activity against sarcoma [18]. The open method for HIPEC was used with vigorous manipulation of all abdominal and pelvic structures to add a mechanical removal of sarcoma cells to the possible effects of hyperthermic chemotherapy [19]. The stapled colorectal anastomosis was performed with a second layer of sutures so that a diverting ileostomy was avoided [20]. The abdomen was closed with non-resorbable suture using small, closely spaced stitches through the linea alba [21].

The patient was discharged on her 14th postoperative day in the absence of adverse events. A readmission of 6 and 24 days was required for failure to thrive. No systemic treatments were recommended. By CT she remains free of disease and with a normal quality of life in June of 2019.

Pathology report showed a low grade myxoid sarcoma with epithelioid morphology in all specimens.

### 3.2 Patient 2

In 2007, a 45-year-old woman experienced heavy menstrual bleeding and lower abdominal pain. A large uterine fibroid was diagnosed. It was laparoscopically resected along with the uterus and then morcellated to allow extraction. Pathology report showed a smooth muscle neoplasm with minimal atypia.

In 2010, the patient complained of abdominal pain and a second laparoscopic resection of what was thought to be “extrauterine leiomyoma” was performed.

**Fig. 3:**
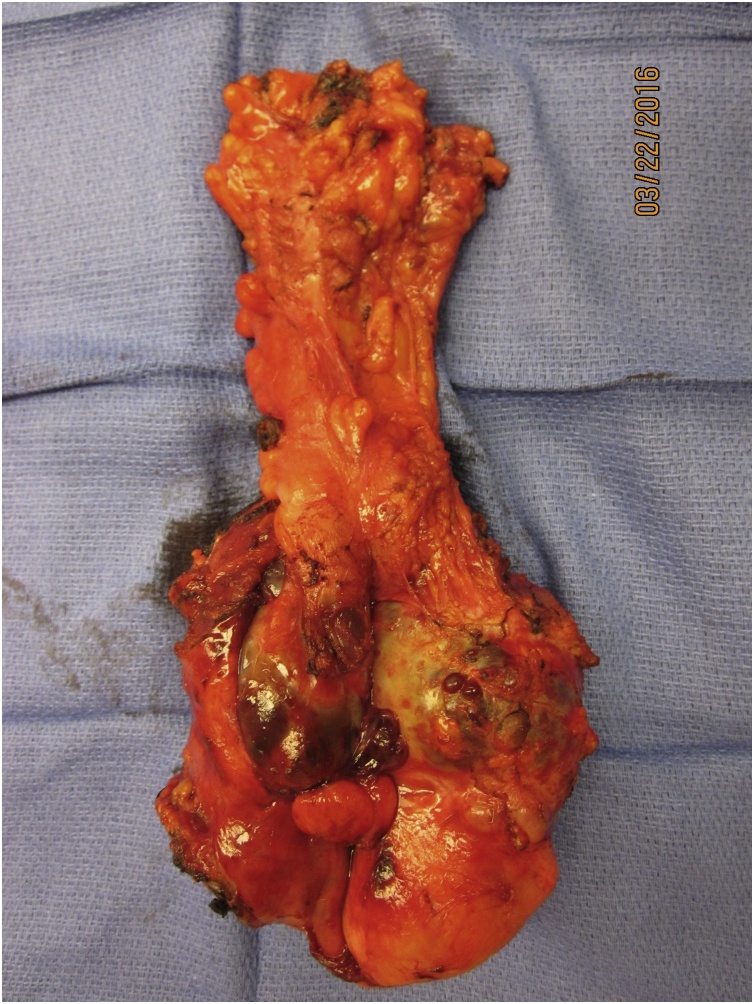
A–F. CT cuts through the abdomen and pelvis showing multiple sarcoma nodules marked by arrows. Nodular masses were seen beneath the right hemidiaphragm (A), at the gallbladder fossa and taile of the spleen (B), filling the right paracolic fossa (C), in the mid-abdomen at the root of the mesentery (D), filling the false pelvis (E), and at the dome of the bladder (F).

In June 2014, abdominal discomfort was experienced especially while exercising. A CT was performed which showed multiple masses of variable size located throughout the abdomen and pelvis (Fig. 3A– F). CT identified 8 masses but no disruption of gastrointestinal or urinary tract function.

In July 22, 2014, the patient underwent a 10-h cytoreductive surgical procedure. At abdominal exploration the PCI was estimated at 20 [16]. All nodules seen on CT were identified and other 1–2 cm nodules associated with the appendix and ovaries were present. The nodule from the undersurface of the right hemidiaphragm was resected using a subphrenic peritonectomy without entrance into the right thorax [17]. Gallbladder and appendix were resected to clear smaller nodules. The large masses were resected using peritonectomy of parietal and visceral peritoneal surfaces. Deep dissection into bowel mesentery was required but no bowel resections were necessary.

After all tumor masses were resected, HIPEC using chemotherapy agents specific for response to sarcoma were used for a 90-minute chemotherapy wash [18]. Extensive agitation was used with the open method to add the mechanical effects of extensive irrigation to the cytotoxic effects of the chemotherapy [19,22].

The patient was discharged from the hospital on her 14th postoperative day in the absence of adverse events. Histopathology showed low grade leiomyosarcoma in all specimens. As of June 2019, the patient remains clinically and radiologically disease-free.

## 4 Discussion

These two patients with extensive sarcomatosis are currently free of disease at 39 and 61 months following definitive cytoreduction plus HIPEC. Further follow-up is indicated but at this point in time their prognosis is favorable for long-term disease-free survival. There were clinical features of both these patients that suggest a long-term success. There was a long time interval from first intervention to definitive cytoreduction without a loss of gastrointestinal or urinary tract function. No ascites was present. Despite the large volume of disease in both patients, these clinical features suggest a low or moderately aggressive disease process.

Radiologically, there were findings to suggest a favorable outcome. No concerning radiologic features were identified [23]. The masses were discrete nodules that were seen to push into the bowel rather than infiltrate. The masses were spherical rather than layering out on the peritoneal surfaces. Despite the fact that some of the masses were large and occupied a significant amount of the peritoneal space, large and small bowel function were preserved. Also, by CT the large masses showed minimal or no necrosis.

The results of surgery suggested a favorable long-term outcome. In both patients a long surgical procedure was required but all visible disease could be resected. A complete cytoreduction in sarcoma or other peritoneal surface malignancies is associated with a more favorable prognosis [4,24]. Using peritonectomy to gain a margin of resection a narrow but with HIPEC adequate margin was possible. Although smaller nodules not seen on CT were present around the appendix, these could be completely resected with appendectomy. Multiple small nodules of sarcomatosis widely distributed within the abdomen and pelvis were not encountered. Complete visible clearance of the sarcoma nodules was possible and the cytoreduction was scored as complete [16].

Absolute containment of microscopic disease could not be assured in either of these two patients. For that reason a warm chemotherapy washing using the open HIPEC technique was used [22]. Also, vigorous manipulation of all abdominal contents was performed not only to uniformly distribute the heat and chemotherapy solution, but also to mechanically remove spilled cancer cells as much as possible [19]. The efficacy of this intraperitoneal chemotherapy treatment has not been established by a randomized trial. Perhaps adding HIPEC to the management of these and similar patients is reasonable until more definitive data becomes available. The HIPEC must be performed safely in this clinical situation.

Knowledge of a moderate to low grade of sarcoma for these two patients was important in their selection for the cytoreductive surgical procedure with HIPEC. This information suggested that a complete removal of all disease would be possible even though the extent of cancer was large. Data from the peritoneal metastases literature supports the complete cytoreduction of large volumes of tumor widely disseminated throughout the abdomen and pelvis if the grade of cancer is low [24]. In contrast, if the malignancy is aggressive complete cytoreduction is possible only with a limited extent of disease. In the absence of concerning radiologic features a major cytoreduction should usually be successful if the malignancy is minimally aggressive even if it is of great extent [4,23]. Only complete cytoreduction is associated with long-term benefit. Morbidity and mortality for an extensive cytoreduction is considerable. Therefore, the surgeon must make sure the risks are commensurate with the benefits.

A sarcoma-specific HIPEC was used to definitively treat all abdominal and pelvic surfaces following the complete cytoreduction [18]. This attempt to clear all surfaces of small amounts of residual sarcoma cells, not appreciated by visual inspection, was performed prior to placing sutures to perform an anastomosis or close the abdominal wall. This timing of the HIPEC is to prevent the entrapment of sarcoma cells within suture lines or abdominal incision.

There may be a strong rationale for long-term success when complete cytoreductive surgery and HIPEC are used to treat minimally invasive malignancies with peritoneal metastases. A small volume of residual disease may exist even after the most meticulous complete cytoreductive surgery. However, in contrast to high grade peritoneal metastases with a complete cytoreduction, the minimally invasive sarcoma does not invade deeper into peritoneal surfaces than the HIPEC penetrates. The high grade malignancies can be made visibly free of peritoneal tumors, but invasion of the sarcoma into the tissues beyond the depth of penetration of HIPEC allows disease progression at a later time. Although this is often referred to as recurrent sarcoma it is, in actuality, persistence and then progression of disease. Patients with other low grade tumors such as mucinous appendiceal metastases and low malignant potential ovarian neoplasms with peritoneal metastases may have long term benefits for the same reason [25,26].

A second explanation for the success of cytoreductive surgery and HIPEC for low grade sarcomatosis is near complete absence of systemic metastases. Liver and lung metastases are unfortunately common with an invasive sarcoma. If systemic metastases occur the benefits of eradication of sarcomatosis will not be appreciated. A lack of systemic metastases with low grade sarcoma allows the benefits of sarcomatosis eradication to be recognized.

## Funding

Data management and secretarial support provided by Foundation for Applied Research in Gastrointestinal Oncology.

## Ethical approval

MedStar Health Institutional Review Board has determined that a case report of less than three (3) patients does not meet the DHHS definition of research (45 CFR 46.102(d)(pre-2018)/45 CFR 46.102(l)(1/19/2017)) or the FDA definition of clinical investigation (21 CFR 46.102(c)) and therefore are not subject to IRB review requirements and do not require IRB approval.

## Consent

Written informed consent was obtained from the patients for publication of this case report and accompanying images. Copies of the written consents are available for review by the Editor-in-Chief of this journal on request.

## Author’s contribution

Paul H. Sugarbaker, MD: study concept or design, data collection, data analysis or interpretation, writing the paper

## Registration of research studies

This study was registered as a case report on the www.researchregistry.com website with UIN 4889.

## Guarantor

Paul H. Sugarbaker, MD.

## Provenance and peer review

Not commissioned, externally peer-reviewed.

## Conflicts of interest

The author has no conflicts of interest to declare.
